# Mapping the Scene and Object Processing Networks by Intracranial EEG

**DOI:** 10.3389/fnhum.2020.561399

**Published:** 2020-10-09

**Authors:** Kamil Vlcek, Iveta Fajnerova, Tereza Nekovarova, Lukas Hejtmanek, Radek Janca, Petr Jezdik, Adam Kalina, Martin Tomasek, Pavel Krsek, Jiri Hammer, Petr Marusic

**Affiliations:** ^1^Department of Neurophysiology of Memory, Institute of Physiology, Czech Academy of Sciences, Prague, Czechia; ^2^National Institute of Mental Health, Prague, Czechia; ^3^Department of Circuit Theory, Faculty of Electrical Engineering, Czech Technical University in Prague, Prague, Czechia; ^4^Department of Neurology, Second Faculty of Medicine, Charles University and Motol University Hospital, Prague, Czechia; ^5^Department of Neurosurgery, Second Faculty of Medicine, Charles University and Motol University Hospital, Prague, Czechia; ^6^Department of Paediatric Neurology, Second Faculty of Medicine, Charles University and Motol University Hospital, Prague, Czechia

**Keywords:** stereoencephalography, high-frequency gamma activity, parahippocampal place area, lateral occipital complex, human brain, visual processing, scenes, objects

## Abstract

Human perception and cognition are based predominantly on visual information processing. Much of the information regarding neuronal correlates of visual processing has been derived from functional imaging studies, which have identified a variety of brain areas contributing to visual analysis, recognition, and processing of objects and scenes. However, only two of these areas, namely the parahippocampal place area (PPA) and the lateral occipital complex (LOC), were verified and further characterized by intracranial electroencephalogram (iEEG). iEEG is a unique measurement technique that samples a local neuronal population with high temporal and anatomical resolution. In the present study, we aimed to expand on previous reports and examine brain activity for selectivity of scenes and objects in the broadband high-gamma frequency range (50–150 Hz). We collected iEEG data from 27 epileptic patients while they watched a series of images, containing objects and scenes, and we identified 375 bipolar channels responding to at least one of these two categories. Using K-means clustering, we delineated their brain localization. In addition to the two areas described previously, we detected significant responses in two other scene-selective areas, not yet reported by any electrophysiological studies; namely the occipital place area (OPA) and the retrosplenial complex. Moreover, using iEEG we revealed a much broader network underlying visual processing than that described to date, using specialized functional imaging experimental designs. Here, we report the selective brain areas for scene processing include the posterior collateral sulcus and the anterior temporal region, which were already shown to be related to scene novelty and landmark naming. The object-selective responses appeared in the parietal, frontal, and temporal regions connected with tool use and object recognition. The temporal analyses specified the time course of the category selectivity through the dorsal and ventral visual streams. The receiver operating characteristic analyses identified the PPA and the fusiform portion of the LOC as being the most selective for scenes and objects, respectively. Our findings represent a valuable overview of visual processing selectivity for scenes and objects based on iEEG analyses and thus, contribute to a better understanding of visual processing in the human brain.

## Introduction

Scene and object visual perception form the fundamentals of our understanding of the world around us. Scenes can be understood as a view of space within which we can move and act, while objects are individual parts of these scenes that we can manipulate. Early functional imaging studies revealed preferential responses to scenes in brain areas along the collateral sulcus, designated the parahippocampal place area (PPA; Aguirre et al., 1998; Epstein and Kanwisher, 1998; Ishai et al., 1999). Another scene-responsive region was described in the retrosplenial-medial parietal region (O’Craven and Kanwisher, 2000), named the retrosplenial complex, or medial place area (MPA) to avoid confusion with the retrosplenial cortex (Epstein and Baker, 2019). Preferential responses to scenes have also been described in the occipital cortex (OC), in the proximity of the transverse occipital sulcus (Nakamura et al., 2000; Hasson et al., 2003). Originally, this region was labeled anatomically as the TOS by the sulcus name, but it was later renamed the occipital place area (OPA) to stress its functional localization (Dilks et al., 2013). In contrast, visual perception of everyday objects evokes a larger hemodynamic response than the perception of scrambled objects in the lateral OC extending to the posterior lateral and the basal temporal regions. This area was originally described as the lateral occipital complex (LOC; Malach et al., 1995), and later subdivided into two functional portions (Grill-Spector et al., 1999): the posterior (labeled LO), and the anterior, localized in the posterior fusiform gyrus (labeled pFs). Nevertheless, scene and object perception are highly interconnected; object perception is dependent on scene context, and the incorporated objects influence scene recognition (Brandman and Peelen, 2017).

While some of the regions responding selectively to scenes and objects are well documented in functional imaging studies, they are only partially supported by direct intracranial electroencephalogram (iEEG) recordings with high (milliseconds) temporal resolution and, in the implanted areas with a high anatomical resolution. The selectivity for scenes, around 250–300 ms after stimulus presentation, has been confirmed in the parahippocampal gyrus for both local field potentials and single-unit activity (Mormann et al., 2017), and also along the collateral sulcus near the parahippocampal/lingual boundary in the broadband gamma range (Bastin et al., 2013a,b). However, confirmation of the scene selectivity of the MPA and OPA, by iEEG analysis is lacking. Nonetheless, selective activity, associated with scene presentation, has been described in the hippocampus for both the firing rate and local field potential (Kraskov et al., 2007). Responses to objects within the fusiform portion of the LOC area (pFs) were described in an early electrocorticography study with a larger N200 component in the inferior lingual, fusiform, and inferior occipital gyri (Allison et al., 1999) and later in an iEEG study for broadband gamma activity (BGA; Vidal et al., 2010). Single unit object-selective activity from the LO, with a delay of about 225 ms after the stimulus, was reported in a recent study using microelectrode grids (Decramer et al., 2019).

Most functional imaging studies focusing on scene and object perception reported the properties of the PPA, MPA, OPA, and LOC areas. However, other brain regions involved in scene and object processing have been identified using specific experimental fMRI designs. Structures of the anterior part of the medial temporal lobe, hippocampus, and parahippocampal gyrus, seem to be more active for a novel, rather than familiar scenes (Rombouts et al., 2001; Köhler et al., 2002). Also, similarly to the PPA area, the anterior hippocampal region showed higher activation for scenes than for objects (Köhler et al., 2002). On the other hand, the naming of unique landmarks seems to be associated with the left temporal pole (Tranel, 2006). Other cortical areas are involved in the visual processing of objects, depending on their type. Passive viewing of familiar tools is connected with higher activity in the premotor cortex and the inferior frontal gyrus (Grafton et al., 1997). The activity of the premotor cortex, together with the middle temporal gyrus and intraparietal sulcus, was increased during the presentation of novel manipulatable objects after training (Weisberg et al., 2006). In contrast, recognition of familiar objects has been associated with higher activity in the inferior frontal gyrus, along the occipitotemporal sulcus and anterior parts of the fusiform and parahippocampal gyri (Bar et al., 2001) and perirhinal cortex (Clarke and Tyler, 2014).

In our study, we aimed to identify the brain networks and anatomical areas facilitating scene and object processing using iEEG. To this end, we examined recordings from 27 epilepsy patients implanted with intracerebral electrodes while they were engaged in a simple visual detection task with stimuli including pictures of scenes and objects. In the analysis, we focused on the (BGA, 50–150 Hz) responses, correlating with both the fMRI BOLD signal (Mukamel et al., 2005; Ojemann et al., 2009) and local neuronal firing rate (Manning et al., 2009; Hammer et al., 2016). We analyzed the iEEG data to identify the category-selective processing within a few hundred milliseconds after stimulus onset and employed the K-means clustering algorithm to group the localization of category-selective responses without any prior neuroanatomical assumptions. Using ROC analysis we evaluated the degree of discrimination between both categories. Our results confirm the scene responding to PPA and object responding to LOC areas, similar to previous iEEG studies (Bastin et al., 2013a; Decramer et al., 2019). Furthermore, we describe electrical activity in two scene-selective areas, the OPA and MPA, not yet reported by electrophysiological studies. Also, our results reveal a much broader network for scene-selective processing in the anterior temporal lobe, as well as for object-selective processing in the parietal, frontal, and temporal cortices.

## Materials and Methods

### Patients and Recordings

Twenty-seven patients (15 women, median age 30 years, range 17–48 years, education level: three primary schools, 20 secondary schools, and three colleges) with drug-resistant epilepsy investigated before epilepsy surgery, were recruited from the Motol Epilepsy Center in Prague. For precise localization of the seizure onset zone, the patients underwent intracranial EEG recordings (iEEG), and stereo encephalography, employing stereotactically implanted multi-contact electrodes. Recording sites were selected solely according to clinical indication with no reference to the presented experiment. This study was approved by the Ethics Committee of Motol University Hospital and all patients gave their informed consent to participate. All patients had normal or corrected to normal vision.

### Electrode Implantation

Eleven to fifteen semi-rigid electrodes per patient were implanted intracerebrally and positioned dependent on the suspected origin of their seizures. Each electrode had a diameter of 0.8 mm and consisted of 8–18 contacts of 2 mm length, 1.5 mm apart (DIXI Medical Instruments). Electrode contacts were identified on patient postimplantation CT and coregistered to preimplantation MRI. The contact anatomical positions were visually verified by an experienced neurologist. The brain was normalized to Montreal Neurological Institute (MNI) space using standard Statistical Parametric Mapping algorithms (SPM 12) and all contacts were localized in the standard MNI space. The iEEG signal was recorded using a video-EEG monitoring system (Natus NicoleteOne in 22 or Natus Quantum in five patients). The data were sampled at 512, 2,048, or 8,000 Hz, using reference electrodes located in the white matter.

### Stimuli and Task

**Figure 1:**
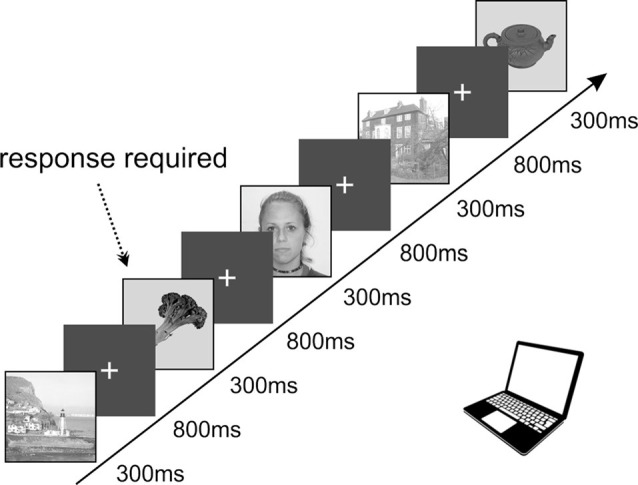
Schematic representation of the timing in our task containing a set of images displaying objects, faces, and spatial scenes presented in a pseudorandom order. The stimuli were organized into groups of five, with a 3-s pauses in-between. The infrequent images of fruits or vegetables (8.3%) requiring active responses were used to keep subjects focused on the presented stimuli. All images were selected from the publicly available databases: objects, fruits, and vegetables from the SUN Database (Xiao et al., 2010), spatial scenes from the Bank of Standardized Stimuli (BOSS; Brodeur et al., 2010) and faces from the lifespan database of adult facial stimuli (Minear and Park, 2004).

All the patients voluntarily participated in a series of experiments focused on visual recognition and spatial orientation. The results we present here were obtained from a task exploring visual recognition of four categories of objects, designed according to the previously published PPA localizer (Vidal et al., 2010; Bastin et al., 2013b). The task lasted approximately 25 min and consisted of 650 pictures in total. We used pictures of three categories: scenes (referred to as “Scenes”), small objects of daily life (referred to as “Objects”), and faces (see Figure 1). This study focuses on responses to Scenes and Objects only. The pictures were selected from the Bank of Standardized Stimuli (BOSS; Brodeur et al., 2010) and the SUN Database (Xiao et al., 2010). To control for a potential decrease in attention, patients were instructed to press a button each time a picture of a fruit or vegetable appeared on the screen (fourth category, visual oddball paradigm). Each category consisted of 100 different pictures (except fruits/vegetables with 25 different pictures), each repeated twice, with a pseudorandom number of other pictures in-between. All stimuli were grayscale squares, 11 cm wide, with normalized average luminance and contrast by ImageMagick^®^ software. Stimuli were presented for a duration of 300 ms every 1,100 ms in blocks of five pictures interleaved by 3-s pause periods to rest the eyes. Patients reported the detection of a target (fruit/vegetable) by pressing the space-bar on a keyboard and were given feedback on their performance (number of correct responses and their average reaction time) after each block. The analysis was only performed on trials in which participants did not press a key.

Visual stimuli were delivered on a 15.6” TFT notebook monitor with a refresh rate of 60 Hz, using the PsychoPy 1.84 environment (Peirce et al., 2019). The monitor was positioned about 60 cm from the subject’s eyes, making the stimuli cover 10° of the visual field. We synchronized stimulus presentation and the EEG recording, using TTL pulses sent to the EEG acquisition parietal cortex (PC) with each stimulus.

### Data Analysis

Time-frequency analyses of the EEG data were performed using a custom package (freely available at https://github.com/kamilvlcek/iEEG_scripts/releases/tag/v1.1.0) in MATLAB 9.4 (Mathworks, Inc.). The data were resampled to 512 Hz unless recorded at this frequency, and channels with obvious artifacts were excluded. From the EEG recording of the whole experiment, bipolar derivations were computed between adjacent electrode contacts to suppress contributions from distant neuronal assemblies and further assumed that the bipolar EEG signals can be considered as originating from a cortical volume centered between the two contacts. We refer to the bipolar contact pairs further as “channels.” The time-frequency analysis was focused on a (BGA, 50–150 Hz). Instantaneous amplitude was estimated using the following procedure (similar to Bastin et al., 2013a): the entire recording dataset was band-pass filtered (third order Butterworth filter, zero phase shift) in consecutive non-overlapping 5 Hz frequency bands in the broad gamma range (e.g., 50–55, 55–60,…, 145–150 Hz). For each band, we extracted the amplitude envelope using a Hilbert transform. The obtained envelope was down-sampled to 64 Hz. For each frequency band, the envelope was then divided by its mean value over the entire recording session, channel-wise, to whiten the EEG power spectrum and compensate for the frequency 1/*f*-power decay of EEG signals (Miller et al., 2009). This yielded 20 amplitude time-series between 50 and 150 Hz (one for each frequency band), which were subsequently averaged together and multiplied by 100 to obtain a single time-series of BGA power for each channel expressed in percent of the mean value. This signal was then epoched into data segments between −200 and 800 ms relative to the stimulus onset. The mean of the prestimulus interval (−50 to 0 ms) was subtracted from each epoch to remove signal changes independent of the respective stimulus. For each channel independently, epochs containing interictal epileptiform discharges identified by a spike detector implemented in MATLAB (Janca et al., 2015) were excluded from further analysis.

The BGA responses were used to identify channels selective for each stimulus category for further analysis, as follows. For all recorded EEG channels, we calculated the average BGA during the prestimulus interval (−200 to 0 ms) for all trials of the respective category and compared it with all time points between 0 and 800 ms post-stimulus using the two-sided Wilcoxon rank-sum test corrected for multiple comparisons across the time dimension and all channels with a false discovery rate (FDR) procedure (Genovese et al., 2002). As a conservative estimate, we used a sliding window of six samples (93.75 ms) with the highest *p*-value. If there was a significant difference at any time point relative to the baseline for a selected stimulus category, the channel was considered as responding to that category. Channels that showed a significant response to any of the two categories (Scenes, Objects) were considered to be “active channels.” After exclusion of channels localized in the white matter or heterotopic cortex or with a response containing obvious artifacts or appearing too late (still increasing at 800 ms, therefore with an impossible to determine magnitude for our epoch length), these channels were subject to further analysis.

To evaluate the differences in response between the two categories, we compared each channel response in both categories for all time points using the same procedure as above. The two-sided Wilcoxon rank-sum tests comparing the response to both categories were computed for all recorded EEG channels and all post-stimulus time points, and again FDR corrected for multiple comparisons across all channels and across the time dimension. A channel with a significant difference in its response to both categories was considered category-selective, either Scene- or Object-selective. The latencies of these effects were compared using two complementary methods. First, we compared the time course of each channel response to both stimulus categories by averaging the response over 100-ms time bins (similar to Bastin et al., 2013a). These means were then analyzed using a three-way repeated measure ANOVA (stimulus category vs. time bins vs. brain region/cluster) with *post hoc* Tukey HSD test and are reported with the effect size (η^2^). Second, we used three measures of the temporal dynamics of the channel selectivity (all in ms): (1) the “time of discrimination” (*tsig*) is the first time point when the difference in response to both categories reached the significance level. (2) The “length of discrimination” (*lensig*) is the total length of significant difference in response to both categories. Finally, (3) the “time of maximal discrimination” (*t90*) is the time when the difference in power change in response to both stimulus categories reached 90% of its maximum for the first time. As this last measure (*t90*) is computed from the difference magnitude, and not time course of significance as *tsig* and *lensig*, it can occasionally give distinct results.

To compare the magnitude of the individual channel responses, we calculated the maximum positive power change for each channel for both stimulus categories. This value is referred to as “response magnitude” in the following text. ANOVA with *post hoc* Tukey HSD test was used to compare this value between groups of channels and is reported with the effect size (*η*^2^). *χ*^2^ was used to test the unequal distribution of channel selectivity between the brain regions. In all statistical tests, we used the significance level of *p* < 0.05.

We used K-means clustering with city-block distance metrics to segment the MNI locations of the category-selective channels, as implemented with the “kmeans” function in Matlab, according to a procedure published previously for iEEG data (Engell and McCarthy, 2014). Using silhouette analysis, we estimated the optimal number of clusters, with all channels being closest to the assigned cluster centroid and most far from others. If these clusters were unstable (i.e., with different centroid positions or different assignment of channels to clusters) over several runs of “kmeans,” we lowered their number until a stable solution was reached. To increase the cluster stability, we implemented a recent seed initialization method (von Luxburg, 2010). Because of the rather low number of category-selective channels, the right and left hemisphere channels were pooled together by using absolute values of MNI “*x*” coordinates. Therefore, each cluster can contain both left and right hemisphere channels.

To assess the response selectivity for individual stimulus categories, we used a receiver operating characteristic (ROC) binary classifier from signal detection theory (Green and Swets, 1966). The area under the curve (AUC) index was estimated from the response size to Scenes and Objects for each time point for each channel. For channels responding more to Scenes than Objects, we evaluated the power to discriminate Scenes from Objects and vice versa.

## Results

### Behavioral Results

The patients mainly responded correctly to fruits or vegetables (an error rate of 5.3 ± 1.9%) and did not respond to other categories (an error rate of 0.78 ± 0.2%). The average response time for fruits or vegetables was 542 ± 13 ms.

### Significantly Activated Channels

**Figure 2:**
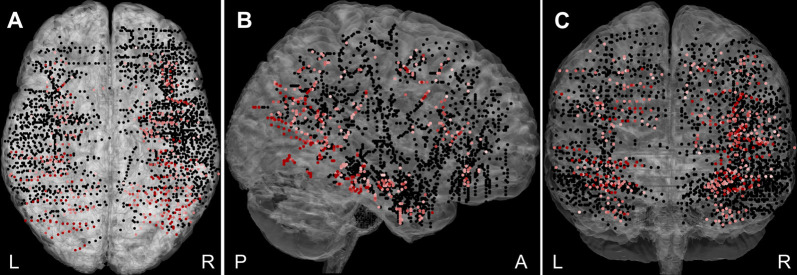
The plot of all 2,707 recorded channels across 27 patients on a standard Montreal Neurological Institute (MNI) brain in **(A)** axial, **(B)** sagittal, and **(C)** coronal plane. The channels responding to Scenes or Objects are plotted in shades of red (higher response magnitude is darker), non-responding in black. The channels were distributed over most of the cortex, but with variable density and excluding the posterior occipital cortex. L, left; R, right; A, anterior; and P, posterior.

Overall, 2,707 bipolar channels (Figure 2) were obtained from the 27 patients, with more recording sites being in the right hemisphere (64%) than the left. A significant response to at least one category, Scenes or Objects, relative to the baseline (−200 to 0 ms, relative to stimulus onset), was identified in 448 (16.5%) channels. Of these, 73 were excluded due to, white matter or heterotopic cortex localization, the response being an artifact, or appearing too late (see “Materials and Methods” section). The remaining 375 channels constitute the basic set for the analysis. Out of these, 71 were labeled “epileptic,” i.e., either located in the seizure onset zone or manifesting high interictal epileptiform activity. To compare epileptic and non-epileptic channels we used two-way ANOVA for the channels, which responded to both Scenes and Objects, with the Scene vs. Object response as repeated measures factor. To compare the response time of the individual channel responses, we calculated the time in ms when the positive power change for both stimulus categories reached 90% of its maximum for the first time. We found no difference in the response magnitude (*F*_(1,175)_ ≤ 0.001, *p* = 0.98, η^2^ < 0.01) or the response time (*F*_(1,175)_ = 1.060, *p* = 0.31, η^2^ < 0.01). Similarly, two-way ANOVA for the channel responding to either Scenes or Objects, with the Scene vs. Object as a factor, did not reveal a significant difference in response magnitude (*F*_(1,194)_ = 0.368, *p* = 0.54, η^2^ < 0.01) nor response time (*F*_(1,194)_ = 1.66, *p* = 0.20, η^2^ < 0.01). Despite the epileptic activity, these channels seemed to be functional and the epileptic activity did not correlate with our visual oddball paradigm. The epileptic channels were therefore included in the analysis. Note, however, that all epochs showing epileptic activity were excluded (see “Materials and Methods” section).

**Figure 3:**
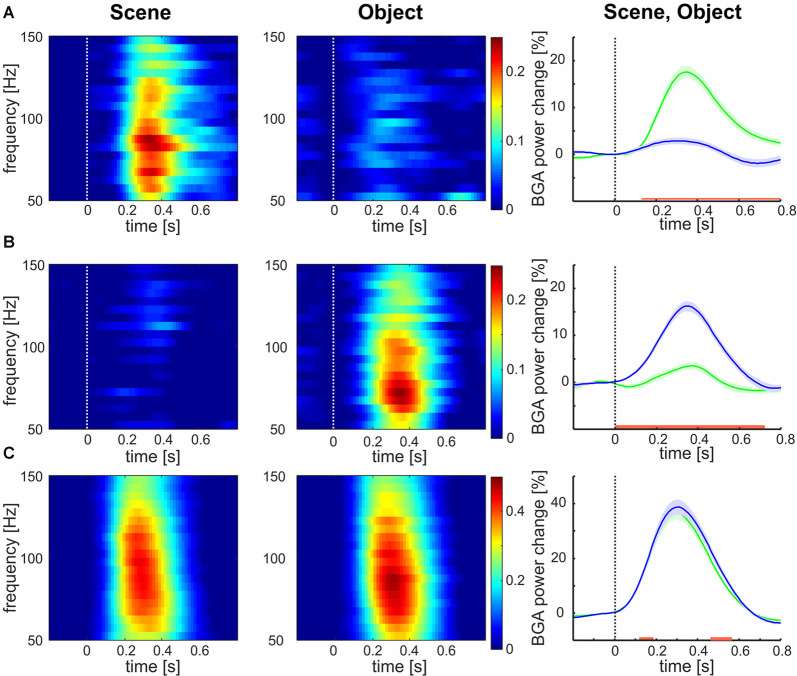
Mean broadband gamma activity (BGA) power responses to both categories for all channels responding to **(A)** Scenes, **(B)** Objects, and **(C)** both. Left two columns: mean over channels. Right column: mean ± SEM over both channels and frequency bands 50–150 Hz, responses to Scenes are in green, responses to Objects are in blue. The red line marks the region of significant difference by false discovery rate (FDR) corrected Wilcoxon signed-rank at *p* < 0.05. Note the different scales for panels **(A,B)** than for panel **(C)**.

Of the 375 channels, relative to the baseline, the highest number of channels (177, 47%) responded to both categories, 123 channels (33%) responded to Objects exclusively and 75 (20%) to Scenes only. The mean responses to each stimulus category are shown in Figure 3. The channels responding to both Scenes and Objects (see Figure 3C) showed larger response magnitude and faster time of discrimination than channels responding only to Scenes (see Figure 3A, magnitude, *t*-test: *t*_(250)_ = 4.58, *p* < 0.001; *tsig*, *t*-test: *t*_(250)_ = 6.57, *p* < 0.001), or only to Objects (see Figure 3B, magnitude: *t*-test: *t*_(298)_ = 6.68, *p* < 0.001; *tsig*, *t*-test: *t*_(298)_ = 8.57, *p* < 0.001). On the contrary, the response magnitude and time of response was similar for channels responding only to Objects (magnitude 43%; time 146 ms) and only to Scenes (magnitude 42%, *t*-test: *t*_(176)_ = 0.47, *p* = 0.63; *tsig* 152 ms, *t*-test: *t*_(176)_ = 1.03, *p* = 0.30). Also, channels responding to both Scenes and Objects responded similarly to both categories (magnitude: Scenes 21%, Objects 20%, *t*-test: *t*_(196)_ = 0.58, *p* = 0.55; *tsig* : Scenes 245 ms, Objects 244 ms, *t*-test: *t*_(196)_ = 0.04, *p* = 0.97).

**Figure 4:**
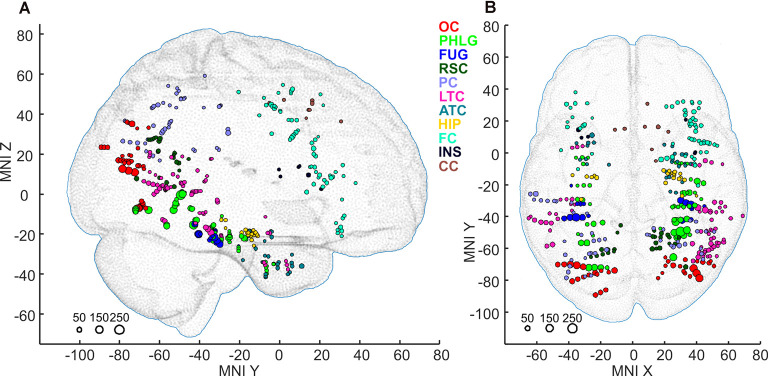
Positions of all 375 active channels responding to Scenes and/or Objects plotted in standard MNI brain in sagittal **(A)** and axial **(B)** views. The size of each point corresponds to the maximum magnitude of each channel’s response (to either Scenes or Objects), with the scale at the bottom left in percent signal change. Channels are marked by different colors according to the 11 brain regions. As a background, we used the adult MNI-ICBM152 head model (Dempsey et al., 2015; http://www.ucl.ac.uk/dot-hub).

Subsequently, we mapped the distribution of all these channels to anatomical regions of interest (ROIs) in the cortex. We grouped the anatomical location of the active 375 channels into the following 11 brain regions (see also Figures 4, 9); (1) OC (but without primary visual cortex) including the OPA (36 channels); (2) PHLG—parahippocampal and inferior lingual gyri, including the collateral sulcus and the PPA (57 channels); (3) FUG—fusiform cortex without the lateral bank of the collateral sulcus (17 channels); (4) RSC—retrosplenial cortex, superior lingual gyrus, and precuneus including the MPA (25 channels); (5) parietal cortex, other parts of the superior parietal lobule and inferior parietal lobule (46 channels); (6) HIP—hippocampus (22 channels); (7) LTC—the lateral temporal cortex—superior, middle and inferior temporal gyrus (69 channels); (8) ATC—anterior temporal cortex—amygdala, entorhinal gyrus, temporal pole (28 channels); (9) FC—frontal cortex (61 channels); (10) INS—insula brain region (six channels); and (11) CC—cingulate and paracingulate cortex (eight channels).

**Figure 5:**
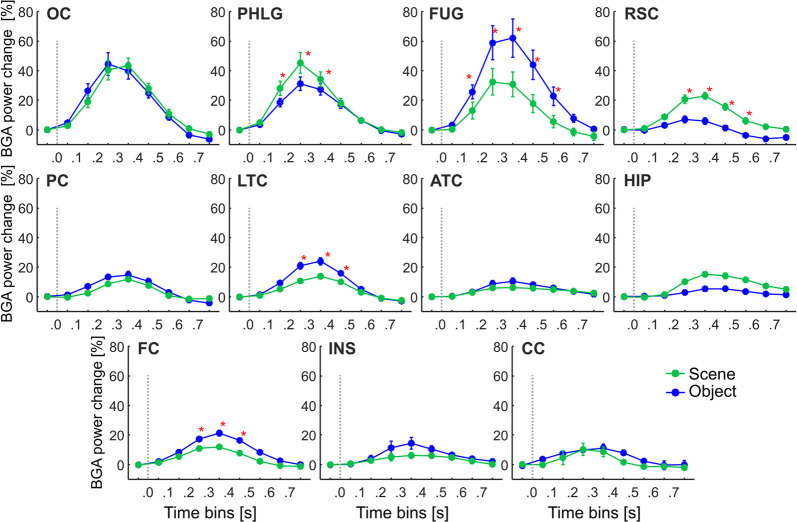
The time course of the group averaged BGA response (mean ± SEM) for all active channels as a function of the anatomically defined brain regions and stimulus type. Significance markers (*) reflect the difference between the response to Scene and Object in each 100-ms time bin. Gray dotted vertical lines mark the stimulus onset. The *x*-axis ticks mark the centers of the time bins, while the *x-axis* labels mark the boundaries of the time bins.

**Figure 6:**
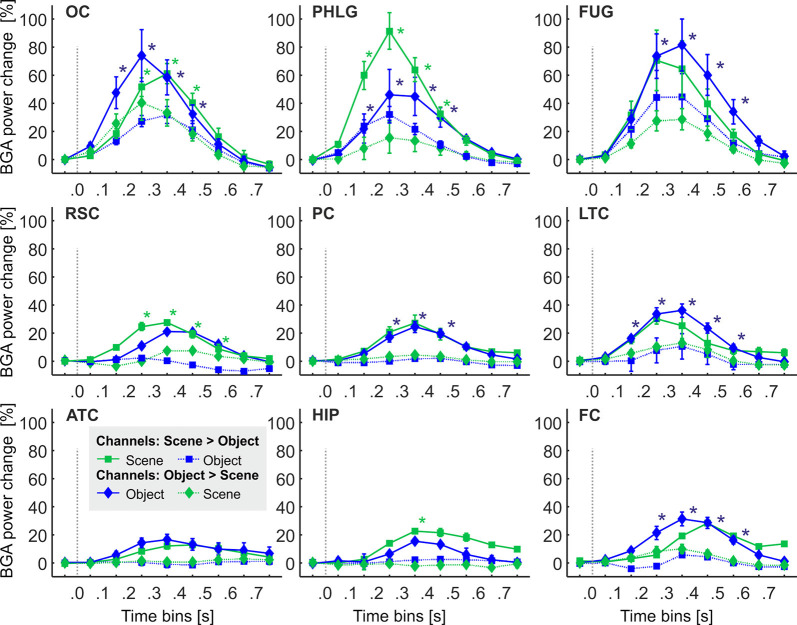
The time course of the group averaged BGA response (mean ± SEM) for Scene-selective (squares) and Object-selective (diamonds) channels, as a function of the anatomically defined brain regions and stimulus type. Solid lines mark responses to the preferred stimulus category, while the dotted line responses to the non-preferred category. Significance markers (*) reflect the difference between the response to Scenes (green) and Objects (blue) in each 100-ms time bin. Same convention as in Figure 5. See also Figure 9 for the numbers of Scenes- and Object-selective channels in each brain region.

**Figure 7:**
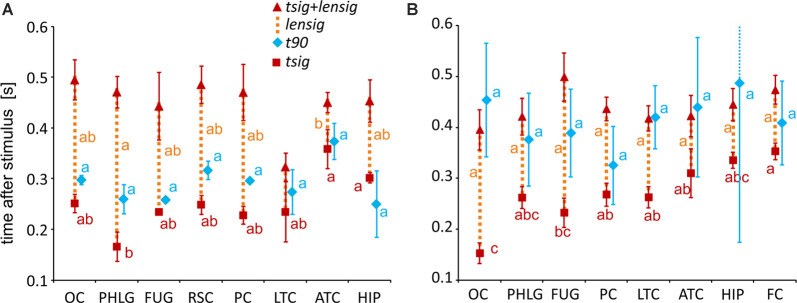
Measures of the timing of the BGA response of Scene- and Object-selective channels sorted by brain region. The time of discrimination (*tsig*) is marked by red squares, while the blue diamonds mark the time of maximal discrimination (*t90*). The length of discrimination (*lensig*) is marked by the dotted orange vertical line stacked in the meantime of discrimination (*tsig*). Therefore, the red triangle marks the time of discrimination plus the length of discrimination (*tsig + lensig*), with the variability of the length of discrimination. All values are mean ± SEM. The same letter (a, b, c) between measures of the same color denotes a lack of significant difference (one-way ANOVA). For example, the time of discrimination of the HIP region (a) was larger than that of the parahippocampal and lingual gyri (PHLG) region (b) but not that of the RSC region (ab). **(A)** Scene-selective channels. **(B)** Object-selective channels.

**Figure 8:**
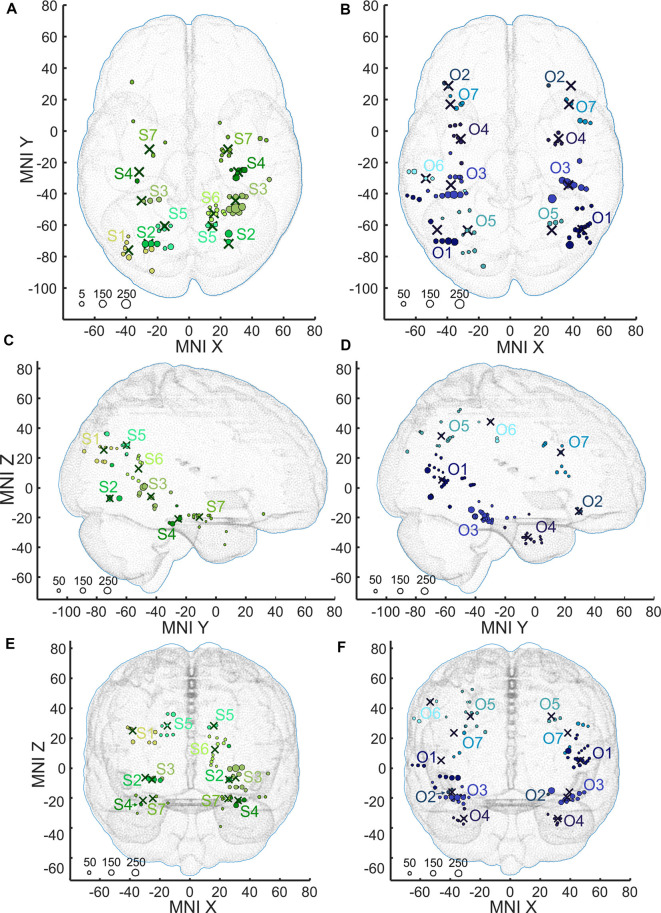
Positions of the channels responding to Scenes more than Objects (S1–S7, shades of green, **A,C,E**) and channels responding to Objects more than Scenes (O1–O2, shades of blue, **B,D,F**) plotted in standard MNI brain in axial **(A,B)**, sagittal **(C,D)**, and coronal **(E,F)** planes. The size of each point corresponds to the maximum magnitude of each channel’s response, with the scale at the bottom left in percent signal change. Channels in each cluster are marked by a different shade of green or blue. Note that centroids of clusters (crosses) are bilaterally symmetrical (see main text). As background, we used the adult MNI-ICBM152 head model (Dempsey et al., 2015; http://www.ucl.ac.uk/dot-hub).

**Figure 9:**
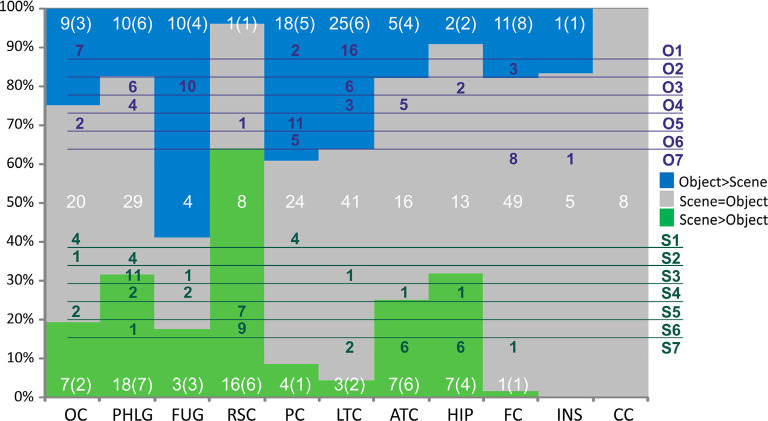
Distribution of channels responding to Scenes more than Objects (green), to Objects more than Scenes (blue) or to both similarly (gray) across the 10 brain regions. Overlaid in white are the numbers of channels for each of these three groups of channels with the number of patients in parentheses. Green and blue letters (right) represent the clusters of Scene- and Object-selective channels. Overlaid in green and blue areas are the numbers of channels in each cluster and their distribution across the brain regions. Legend: OC, occipital cortex; PHLG, parahippocampal and lingual gyri; FUG, fusiform gyrus; RSC, retrosplenial cortex and precuneus; PC, parietal cortex; HIP, the hippocampus; LTC, lateral temporal cortex; ATC, anterior temporal cortex; FC, frontal cortex; INS, insula; CC, cingulate and paracingulate cortex.

These regions differed in the average time course of their response (see Figure 5). A three-way repeated-measures ANOVA (stimulus category vs. time bin vs. brain region) for all channels showed a significant effect of all factors and interactions (the three-way interactions: *F*_(80,2912)_ = 6.71, *p* < 0.001, η^2^ = 0.16), except the main factor of stimulus category. Figure 5 shows the differences in response to both categories for all time bins brain labels, with marked significance. Channels in two regions responded more to Scenes than Objects; in PHLG from 100 to 400 ms and in RSC from 200 to 600 ms (*post hoc* test on the three-way interaction). Channels in the other three regions responded significantly more to Objects than Scenes (FUG, 100–600 ms; LTC, 200–500 ms; and FC, 200–500 ms).

### The Selectivity of Channels to Scenes and Objects and Its Cortical Distribution

To evaluate the channel response selectivity, we directly compared responses to Scenes and Objects, at all time points after the stimulus presentation and within the epoch. Most channels (217, 58%) did not show significant differences between the two categories. However, 92 (25%) channels responded to Objects significantly more than to Scenes and 66 (18%) channels responded significantly more to Scenes than to Objects.

Scene and Object selectivity were not evenly distributed in the brain regions ($\chi_{\left(9,N=158\right)}^{2}$ = 55.40, *p* < 0.001). The Scene-selective channels were localized predominantly in the PHLG (30%), RSC (24%), OC (11%), ATC (11%), and HIP (11%) regions, while the Object-selective channels were mainly in the LTC (27%), PC (2015%), and FC (12%) regions. From another point of view, the HIP (seven of nine channels), RSC (16/17), and PHLG (18/28) region predominantly contained the Scene-selective channels, while more Object-selective channels were observed in the FC (11/12), LTC (25/28), PC (18/22), and FUG (10/13) regions. As the INS region contained only one Object-selective region and the CC region did not contain any category-selective regions, both were excluded from further analyses. Visual inspection of the distribution of Scene- and Object-selective channels in the brain suggested differences in their mediolateral and anteroposterior position (see Figure 8). Analyzing the MNI coordinates, we found that the Object-selective channels were located more laterally (with a larger absolute MNI “*x*” coordinate, *t*_(156)_ = 8.35, *p* < 0.001) and more anteriorly (with a larger MNI “*y*” coordinate, *t*_(156)_ = 2.01, *p* < 0.05) than the Scene-selective channels.

### Temporal Dynamics of Selective Channels in Anatomical Regions

One of the advantages of iEEG analysis is the possibility to analyze the precise temporal dynamics of Scene and Object selectivity. Initial information about the response time course we revealed using analysis of response differences in 100-ms time bins. Two, three-way repeated-measures ANOVAs (stimulus category vs. time bin vs. brain region) for Scene- and Object-selective channels showed a significant effect of all factors and interactions (both three-way interactions: *F*_(56,456/656)_ > 3.3, *p* < 0.001, η^2^ > 0.2). Figure 6 shows the differences in response to both categories for all time bins and brain labels, with marked significance. For the Scene-selective channels (*post hoc* test on the three-way interaction), the first difference in response to Scenes and Objects was in PHLG (100–200 ms), followed by OC and RSC (200–300 ms). In the HIP region, the selectivity emerged later (300–400 ms). As for the duration of the difference in the significance, the longest difference was in the PHLG and RSC region (400 ms) and shortest in the HIP (100 ms). For the Object-selective channels, the *post hoc* test revealed the first significant differences in response to Objects and Scenes in OC, PHLG, and LTC (100–200 ms), followed by PC, FUG, and FC (200–300 ms) regions. The longest difference was in the LTC region (500 ms), followed by PHLG, OC, FUG, and LTC regions (400 ms) and shortest in the PC (300 ms) region.

To specify the time course of category selectivity with a higher time resolution, we used three measures based on our BGA sampling frequency (64 Hz, see Figure 7). First, we compared the time of discrimination (*tsig*) for regions with at least two channels in both channel groups (i.e., excluding RSC and FC). A two-way ANOVA (brain region vs. category) on the time of discrimination did not reveal a significant effect of the category (*F*_(1,114)_ = 0.12, *p* = 0.73), and the interaction was close to significance (*F*_(6,114)_ = 2.11, *p* = 0.06). However, we found differences between the brain regions by two separate one-way ANOVAs for both categories, including the RSC and FC regions (see Table 1 for individual values). The time of discrimination of Scenes from Objects in the PHLG region was earlier than in the MTL and HIP regions (*F*_(7,57)_ = 4.20, *p* < 0.001, *η*^2^ = 0.34, *post hoc* both *p* < 0.05). In contrast, the time of discrimination of Objects from Scenes was the earliest in the OC region and latest in the FC region, later than in the FUG region (*F*_(7,83)_ = 4.21, *p* < 0.001, η^2^ = 0.26, *post hoc* all *p* < 0.05). Concerning the length of discrimination (*lensig*), similar analysis revealed longer period of significant difference in PHLG than ATC regions (*F*_(7,57)_ = 3.28, *p* < 0.01, η^2^ = 0.29, *post hoc* all *p* < 0.01), but no differences between regions in Object-selective channels (*F*_(7,83)_ = 2.16, *p* = 0.05, η^2^ = 0.15, *post hoc* all *p* > 0.05). There were also no differences between the regions in the time of maximal discrimination (*t90*). In general, these results parallel and confirm those using 100-ms time bins, with some exceptions in Object-selective channels. These channels in the FC regions showed a significant difference between the response to Objects and Scenes from the 200–300 ms time bin, but its time of discrimination for Objects was around 350 ms.

**Table 1:** Time of discrimination (*tsig*), time of maximal discrimination (*t90*) and length of discrimination (*lensig*; all in ms, mean ± SEM) for Scene-selective (Scene > Object) and Object-selective (Object > Scenes) channels by brain region (left) and clusters (right).

	Scene > Object				Scene > Object			
	*tsig*	*t90*	*lensig*		Label	*tsig*	*t90*	*lensig*
PHLG	166 ± 29^b^	260 ± 29	305 ± 31^a^	S3	PPA	164 ± 23^ab^	256 ± 14^a^	338 ± 41^b^
PC	228 ± 18	296 ± 4	242 ± 55	S2	pLG	189 ± 35	244 ± 22	191 ± 36
FUG	235 ± 5	258 ± 5	208 ± 66	S4	aCoS	211 ± 33^a^	195 ± 43	128 ± 38^a^
LTC	235 ± 59	274 ± 45	89 ± 28	S5	PCun	215 ± 17	284 ± 14	253 ± 31
RSC	249 ± 19	317 ± 18	236 ± 36	S1	OPA	242 ± 13	294 ± 7	232 ± 28
OC	251 ± 18	298 ± 9	243 ± 39	S6	MPA	307 ± 41^c^	380 ± 43^b^	244 ± 56
HIP	302 ± 10^a^	250 ± 66	152 ± 42	S7	aHip	326 ± 27^ bc^	334 ± 34^b^	125 ± 22^a^
ATC	359 ± 38^a^	374 ± 36	92 ± 19^b^				
	**Object > Scene**				**Object > Scene**			
	***tsig***	***t90***	***lensig***		**Label**	***tsig***	***t90***	***lensig***
OC	153 ± 21^c^	454 ± 112	243 ± 40^a^	O1	LO	215 ± 19^b^	262 ± 14^b^	165 ± 22^a^
FUG	233 ± 29^bc^	389 ± 86	267 ± 48^a^	O3	pFs	255 ± 20	320 ± 14	232 ± 31^a^
PHLG	262 ± 21	377 ± 91	159 ± 36^a^	O5	pAnG	257 ± 31	302 ± 29	152 ± 30^a^
LTC	263 ± 21^ab^	420 ± 62	155 ± 25^a^	O6	aIPS	291 ± 33	344 ± 30	197 ± 51^a^
PC	268 ± 23^ab^	326 ± 77	168 ± 23^a^	O4	aCoS	317 ± 22^a^	348 ± 19^a^	117 ± 25^a^
ATC	310 ± 48^ab^	440 ± 137	113 ± 41^a^	O7	IFS	345 ± 19^a^	378 ± 17^a^	137 ± 32^a^
HIP	336 ± 16	488 ± 313	109 ± 31^a^	O2	OFG	363 ± 28	378 ± 24	52 ± 21^a^
FC	353 ± 16^a^	409 ± 82	121 ± 28^a^					

*The rows are sorted by the mean tsig value. The same letter (a, b, c) between regions and clusters in the same group, or no letter, marks a lack of significant difference. The labels for clusters present approximate locations of cluster centroids but see Figure 9 and Table 2 for more specific cluster localization. aCoS, anterior collateral sulcus; aHip, anterior hippocampus; aIPS, anterior intraparietal sulcus; IFS, inferior frontal sulcus; LO, the posterior portion of the lateral occipital complex; MPA, medial place area; OFG, orbitofrontal gyri; OPA, occipital place area; pAnG, posterior angular gyrus; PCun, precuneus; pFs, anterior portion of the lateral occipital complex; pLG, posterior lingual gyrus; PPA, parahippocampal place area*.

### MNI Based Clustering of Channel Selectivity for Scenes and Objects

**Figure 10:**
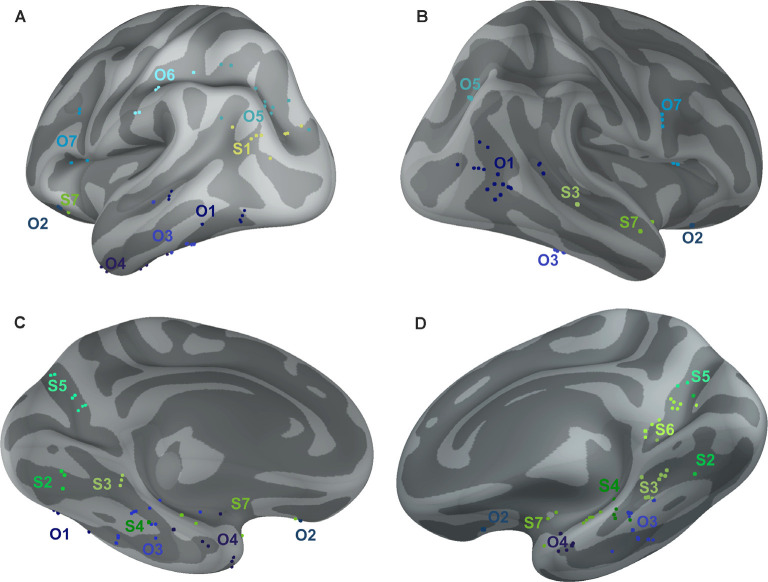
Positions of all channels responding to Scenes more than Objects (S1–S7, shades of green) or to Objects more than Scenes (O1–O7, shades of blue) plotted on inflated FSAverage subject brain (produced using Brainstorm; Tadel et al., 2011). Channels in each cluster are marked by a different shade of green or blue. Lateral **(A,B)** and medial **(C,D)** view of the left **(A,C)** and right **(B,D)** hemisphere.

The 11 anatomical brain regions did not adequately portray the distribution of response selectivity seen (see Figures 8, 9). To further summarize the category-selective channel locations and avoid any prior assumptions of anatomical localization, we used the K-means clustering algorithm (Engell and McCarthy, 2014, see “Materials and Methods” section for more details). The K-means algorithm, explaining 70.9% of the total spatial variance, segmented the 66 Scene-selective channels by their MNI coordinates to seven clusters (marked as S1–S7, see Table 2, Figures 8, 10). The centroids of these clusters were localized to the following structures: the posterior angular and medial occipital gyrus (S1), the posterior collateral sulcus at the junction with the lingual sulcus (S2), the lingual and fusiform gyrus along the middle collateral sulcus (S3), the parahippocampal and fusiform gyrus along the anterior collateral sulcus (S4), the precuneus (S5), the superior lingual gyrus and precuneus next to the retrosplenial region (S6), the anterior hippocampus (S7). Based on the anatomical position and MNI coordinates, the S1 cluster partially overlapped with the OPA, the S3 cluster with the PPA, and the S6 cluster with the MPA.

**Table 2:** List of clusters of Scene- and Object-selective channels.

					MNI coordinates		
Cluster	*N*	L/R	*P*	Brain structures	Abs (*X*)	*Y*	*Z*
S1	8	8/0	2	**ang**(4), mog(4)	38 ± 3	−76 ± 2	24 ± 2
S2	5	3/2	3	**lg**(4), cun(1)	25 ± 11	−72 ± 1	−8 ± 4
S3	13	3/10	5	**fg**(6), lg(3), phg(3), mtg(1)	30 ± 7	−45 ± 2	−7 ± 1
S4	6	1/5	5	fg(2), p**hg**(2), ent(1), hi(1)	32 ± 10	−26 ± 1	−22 ± 2
S5	9	7/2	3	**pcun**(7), cun(2)	15 ± 4	−61 ± 2	27 ± 2
S6	10	0/10	4	**pcun**(4), lg(5), rsc(1)	16 ± 1	−53 ± 1	12 ± 2
S7	15	4/11	9	**hi**(6), ent(3), mtg(2), amg(1), ofg(1), sub(1), tp(1)	25 ± 7	−12 ± 3	−20 ± 1
O1	25	8/17	5	**mtg**(9), mog(5), itg(4), stg(3), ang(2), totz(2)	46 ± 9	−63 ± 2	4 ± 2
O2	3	2/1	3	**ofg**(3)	39 ± 18	29 ± 1	−17 ± 1
O3	24	13/11	9	**fg**(16), itg(5), hi(2), mtg(1)	37 ± 8	−34 ± 1	−20 ± 1
O4	12	7/5	7	itg(3) ,ent(2), fg(2), p**hg**(2), amg(1), tp(2)	31 ± 9	−5 ± 2	−34 ± 1
O5	14	11/3	3	**ang**(10), cun(2), pcun(1), spl(1)	27 ± 6	−63 ± 3	34 ± 2
O6	5	5/0	3	smg(2), **spl**(2), pog(1)	53 ± 6	−30 ± 2	43 ± 3
O7	9	4/5	5	**ifg**(4), prg(3), fop(1), ins(1)	38 ± 13	17 ± 2	23 ± 3

*N, number of channels; L/R, number of channels in the left/right hemisphere; P, number of patients; X, Y, Z, MNI coordinates of the cluster centroids ± SEM. The brain structures column lists anatomical labels for all channels in the cluster, with the number of channels in parentheses, the structure in bold being closest to the cluster centroid: amg, amygdala; ang, angular gyrus; cun, cuneus; ent, entorhinal cortex; fg, fusiform gyrus; fop, frontal operculum; hi, hippocampus; ifg, inferior frontal gyrus; ins, insula; itg, inferior temporal gyrus; lg, lingual gyrus; mfg, middle frontal gyrus; mog, middle occipital gyrus; mtg, middle temporal gyrus; ofg, orbitofrontal gyri; pcun, precuneus; phg, parahippocampal gyrus; pog, postcentral gyrus; prg, precentral gyrus; rsc, retrosplenial cortex; sfg, superior frontal gyrus; smg, supramarginal gyrus; spl, superior parietal lobule; stg, superior temporal gyrus; sub, subiculum; totz, temporo-occipital transition zone; tp, temporal pole*.

Similarly, we used the K-means algorithm to further specify the locations of the 92 Object-selective channels. The algorithm segmented these channels to seven clusters (marked as O1–O7, see Table 2, Figures 8, 10), explaining 70.7% of the total spatial variance. The centroids of these clusters were localized in: around the posterior inferior temporal sulcus (O1), the orbitofrontal gyrus (O2), area around the anterior end of the collateral sulcus (O3), the anterior part of the fusiform gyrus (O4), the posterior part of the angular gyrus (O5), near the anterior intraparietal sulcus (O6) and near the inferior frontal sulcus (O7). The clusters O1 and O3 partially overlapped with the LOC area, it is posterior (LO), and anterior (pFs) portions, respectively (but see “Discussion” section).

#### Temporal Dynamics of Selective Channels in MNI Based Clusters

**Figure 11:**
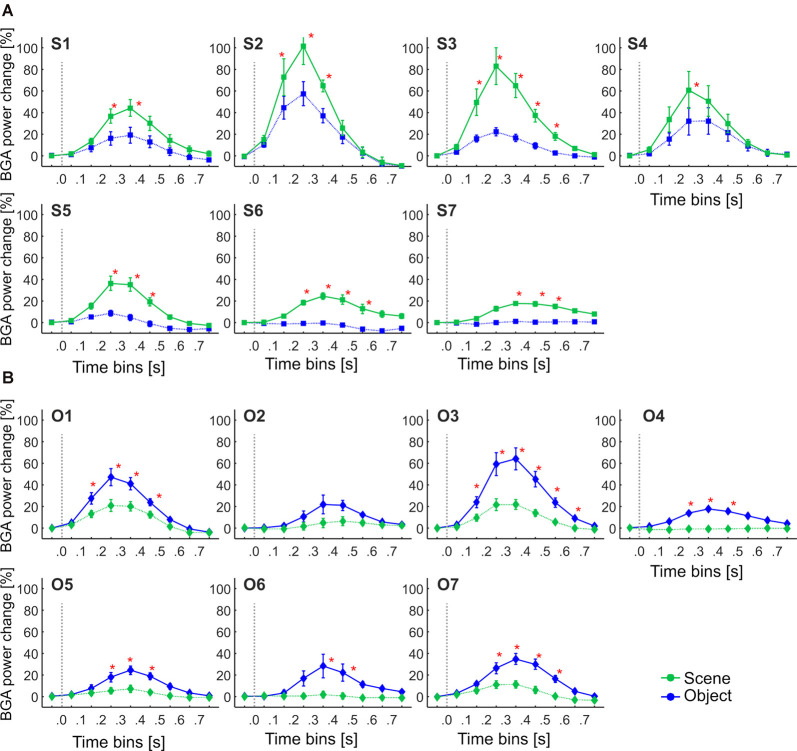
The Time course of the group averaged BGA response (mean ± SEM) for Scene-selective and Object-selective channels, as a function of the MNI based clusters and stimulus type. Significance markers (*) reflect the difference between the response to Scenes (green) and Objects (blue) in each 100-ms time bin. Same convention as in Figure 5. **(A)** Clusters of Scene-selective channels. **(B)** Clusters of Object-selective channels.

We aimed to compare the temporal characteristics of the Scene and Object selectivity in the clusters with the anatomically defined regions. Similarly to brain regions above, we started with the analysis of response differences in 100-ms time bins. Two three-way repeated-measures ANOVAs (stimulus category vs. time bin vs. cluster) for Scene- and Object-selective channels showed a significant effect of all factors and interactions (both three-way interactions: *F*_(48,472/680)_ > 3.7, *p* < 0.001, η^2^ > 0.2). The differences in response to both categories for all time bins and brain labels, with marked significance, can be seen in Figure 11. For the Scene-selective channels, the *post hoc* test on the three-way interaction revealed the first difference in response to Scenes and Objects in S2 and S3 clusters (100–200 ms), followed by S1, S4, S5, and S6 clusters (200–300 ms), with the last cluster S7 (300–400 ms). The cluster with the longest difference between both categories was S3 (500 ms), while the shortest one was cluster S4 (100 ms). For the Object-selective channels, the *post hoc* test revealed the first significant differences in response to Objects and Scenes in clusters O1 and O3 (100–200 ms, followed by O4, O5, and O7 (200–300 ms), with the last cluster O6 (300–400 ms). The cluster with the longest difference between categories was O6 (600 ms), followed by O1 and O7 (400 ms).

**Figure 12:**
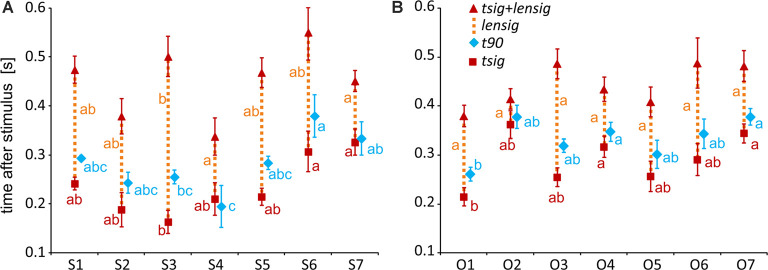
Measures of the timing of the BGA response of Scene- and Object-selective channels, sorted by clusters. **(A)** Clusters of Scene-selective channels. **(B)** Clusters of Object-selective channels. Same convention as in Figure 7.

To specify, with a higher time resolution, how the category selectivity develops in clusters, we again used three measures based on our BGA sampling frequency (64 Hz, see Figure 12). We compared them by separate one-way ANOVAs for both groups of clusters (see Table 1 for individual values). The time of discrimination (*tsig*) of Scenes from Objects in the S3 cluster was earlier than in the S6 and S7 clusters (*F*_(6,59)_ = 5.28, *p* < 0.001, η^2^ = 0.35, all *post hoc* *p* < 0.01). The length of discrimination (*lensig*) was longer in the S3 cluster than in the S4 and S7 clusters (*F*_(6,59)_ = 4.61, *p* < 0.001, η^2^ = 0.32, all *post hoc* *p* < 0.05). The time of maximal discrimination (*t90*) was shorter in S4 cluster than S6 and S7 clusters and in S3 than in S6 cluster (*F*_(6,59)_ = 3.58, *p* < 0.005, η^2^ = 0.27, all *post hoc* *p* < 0.05). In Object-selective channels, the time of discrimination (*tsig*) was the earlier in the O1 than in O7 cluster (*F*_(6,85)_ = 3.72, *p* < 0.005, η^2^ = 0.21, both *post hoc* *p* < 0.05). The length of discrimination (*lensig*) was similar in all clusters (*F*_(6,85)_ = 2.22, *p* = 0.05, η^2^ = 0.14, no *post hoc* *p* < 0.05), while the time of maximal discrimination (*t90*) was shorter in O1 cluster than O4 and O7 clusters (*F*_(6,85)_ = 4.18, *p* < 0.001, η^2^ = 0.23, both *post hoc* *p* < 0.05).

These results again closely parallel and confirm the results from the analysis using 100-ms time bins, with some exceptions in object clusters. The O7 cluster showed a significant difference between the response to Objects and Scenes from the 200–300 ms time bin, earlier than cluster O6, but its time of discrimination was around 350 ms, while in cluster O6 the *tsig* was below 300 ms. Besides, cluster O3 showed the longest difference in response to both stimulus categories, 400 ms longer than cluster O6, but there were no differences in the length of discrimination (*lensig*) between the clusters.

Interestingly, we found more diverse measures of temporal dynamics in the MNI based clusters than in the anatomical brain regions. The time course in the S3 cluster, overlapping the PPA area, was similar to the PHLG region, with an early start and long discrimination between Scenes and Objects. This discrimination started late with a late maximal difference in the cluster S6, with a centroid near the retrosplenial region, but these differences we did not find in the RSC region, including the retrosplenial cortex and precuneus. The time of discrimination in cluster S7, with a centroid near the anterior hippocampus, was late, similarly to the HIP region, but with also the late time of maximal discrimination, which was not paralleled in the HIP region. Also, the cluster S4, with a centroid near the anterior collateral sulcus, showed the fastest time of maximal discrimination and short time of discrimination of Scenes from Objects, with no similar characteristics in any of the anatomical regions. Concerning the Object clusters, we found a fast time of discrimination and time of maximal discrimination in cluster O1 near the posterior inferior temporal sulcus, partially overlapping with the LO area, similarly to the OC region (but with no *t90* difference). Late discrimination was also found in the O7 cluster with a centroid near inferior frontal sulcus, paralleled in the FC region, which, however, included more channels. The cluster O4, with a centroid near anterior fusiform gyrus, showed late category discrimination, not different from the O7 cluster, in contrast to the FUG region with faster category discrimination than the FC region. Finally, the cluster O3 near middle fusiform gyrus, partially overlapping with the pFs area, shower a very long time of difference in response to both stimulus categories in the time bins analyses, with no such long time of difference in any of the anatomical regions.

### ROC Analysis of the Stimulus Categories Discrimination

**Figure 13:**
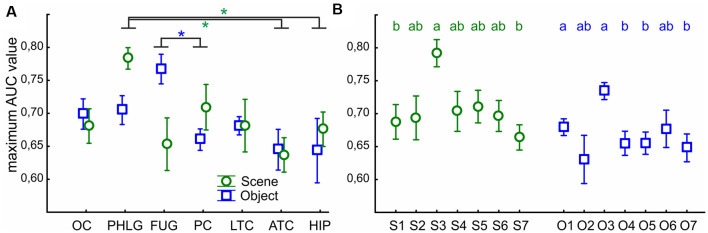
The average maximal AUC values of category-selective channels for Scenes (green circles) and Objects (blue squares). Error bars represent SEM across channels. **(A)** Across the seven analyzed brain regions (see Figure 9 for their list). Green and blue asterisks represent significant differences in the brain region factor (two-way ANOVA at *p* < 0.05) in Scene- and Object-selective channels, respectively. Category discrimination was better for channels in the FLPG region than for the channels in the ATC and FC regions. **(B)** Across the channels with the S1–7 and O1–7 clusters. Similarly to Figure 7, the same letter (a, b, c) between clusters of the same color marks a lack of significant difference.

Finally, we were interested in how reliably we could distinguish if the stimulus was Scene or Object from the single-trial individual channel responses. To this end, we used a ROC analysis to illustrate how well the responses of the two categories were separated, for a series of BGA magnitude thresholds (for a similar procedure see Bastin et al., 2013a). The ROC area under the curve (AUC) is a summary measure of the separation across all thresholds levels. We computed the AUC values for all post-stimulus time samples, for the separation of Scenes from Objects, as well as Objects from Scenes, and compared the maximal AUC values between the seven brain regions with more than two Scene- as well as Object-selective channels. The two-way ANOVA (brain region vs. category) revealed significant differences between the brain regions (*F*_(76,114)_ = 3.83, *p* < 0.005, η^2^ = 0.17), and significant interaction (*F*_(76,114)_ = 2.61, *p* < 0.05, η^2^ = 0.12) with no differences between categories (Figure 13A). The *post hoc* test of the brain region factor showed that category discrimination was better for channels in the PHLG region than for the channels in the ATC and HIP regions (all *p* < 0.05) in Scene-selective channels. In contrast, the FUG regions shower better category discrimination than PC (*p* < 0.05) in Object-selective channels. Then, similarly, we also compared the maximal AUC values for the Scene and Object clusters, using two independent one-way ANOVAs (Figure 13B). The discrimination of Scenes from Objects was better for channels in the S3 cluster than in clusters S1 and S7 (*F*_(6,59)_ = 3.81, *p* < 0.005, η^2^ = 0.30, both *post hoc* *p* < 0.05). Similarly, the discrimination of Objects from Scenes was better for channels in the O3 cluster than channels in any other Object cluster (*F*_(6,85)_ = 4.28, *p* < 0.001, η^2^ = 0.23, *post hoc* all *p* < 0.05), except O2 and O6.

This good discrimination corresponds well with the position of the S3 and O3 clusters near the PPA and the anterior portion of the LOC (pFs), respectively. In parallel, the PHLG and FUG regions showed the best discrimination for Scenes and Objects, respectively.

## Discussion

Our study provides a broad survey of the human cortex, searching for regions that respond in a category-selective fashion to scenes or objects with BGA power increase. We did not restrict our analysis to previously identified category-selective ROI, but instead tested all implanted areas for any scene or object-selective regions, whether previously identified or not. Our results reveal much broader brain networks involved in scene and object processing than previously reported from functional imaging studies with similar experimental designs. Besides the visual perception areas in the ventral stream, we found significant activity in areas previously reported to be associated with scene novelty, scene construction, object recognition, and object tool use. Taking advantage of the fast temporal resolution of iEEG, we used two complementary methods to analyze the time course of discrimination of objects from scenes of vice versa. Employing ROC analysis, we also showed how reliably the analyzed areas discriminate between scenes and objects.

While almost half of the active channels responded to both categories with increased BGA power, a significant proportion of them was selective for either scenes or objects. Channels responding to scenes more than objects comprised 18% of all active channels. Most functional imaging studies have defined the PPA, MPA, and OPA as regions selective for the scenes and landscapes when contrasting their responses to object stimuli (Epstein and Kanwisher, 1998; Nakamura et al., 2000; O’Craven and Kanwisher, 2000). In our study, using intracranial EEG data, we confirmed these three regions to be scene-specific. Most of our scene-selective channels were localized in the PHLG region, in the RSC, and in the OC regions. However, we also found numerous scene-selective channels in other, previously unreported, brain areas, especially in other parts of the temporal lobe (the HIP and ATC regions), with most channels in the hippocampus being selective for scenes. Besides, scene-selective channels were also localized in the parietal, frontal, and lateral temporal cortices.

Channels selective for objects constituted 25% of all active channels. Their position was generally more lateral and anterior compared to the scene-selective channels; most were found in the LTC, PC, and FC regions, but object-selective channels were also apparent in the FUG, PHLG, and OC regions. This distribution corresponded with the results of another human intracranial study (Vidal et al., 2010). The object selectivity in the LTC and FUG regions overlapped with the LOC area, defined by functional imaging studies.

### Areas Selective for Scenes

To further summarize the channel locations, we segmented them into spatially defined clusters, seven with both scene-selective and object-selective channels, and identified the locations of the cluster centroids.

The first scene-selective region to be described was the PPA (Aguirre et al., 1998; Epstein and Kanwisher, 1998), which typically includes portions of the posterior parahippocampal, anterior lingual, and medial fusiform gyri (Epstein and Baker, 2019), along the collateral sulcus. Our S3 cluster was localized to an area with similar MNI coordinates to the PPA recently published location (Spiridon et al., 2006). It almost completely included channels only in the PHGL region. According to our ROC analysis, the degree of discrimination of scenes from objects was largest in this cluster, approaching 0.8. Another functionally defined scene-selective area, the MPA, was described near the cingulate gyrus (O’Craven and Kanwisher, 2000), mostly comprising the retrosplenial cortex and the anterior precuneus. These data agree with the localization of our S6 cluster in the RSC region, specifically in the precuneus and the superior part of the lingual gyrus near the retrosplenial cortex, along the banks of the parietal-occipital sulcus. The third most commonly reported scene-selective region is the OPA in the occipital lobe (Nakamura et al., 2000; Hasson et al., 2003), typically near the transverse occipital sulcus. Originally labeled the TOS, it was later renamed the OPA (Dilks et al., 2013) to emphasize its functional localization. Our S1 cluster was localized to a similar area, in the middle occipital gyrus, also encompassing channels in the posterior angular gyrus. It included channels from OC and also PC regions. Surprisingly, the degree of discrimination (i.e., the average maximal AUC value) of scenes from objects in this cluster was below 0.7, significantly lower than in the cluster S3.

Many scene-selective channels in our study were localized to the HIP region, forming about half of the S7 cluster, together with the ATC region. The hippocampus has not been routinely described as a scene-selective region; however, its association with scene processing is well known. An early PET study showed anterior hippocampal activation in response to novel scenes and also a larger response to scenes than to objects in a scene-learning task (Köhler et al., 2002). Another element of the hippocampus, the presubiculum/parasubiculum, was also found to be active during scene recall and imagination (Zeidman et al., 2015). Selectivity for spatial layouts has been described for about 30% of hippocampal neurons (Kreiman et al., 2000). The scene construction theory even proposes the main hippocampal function to be the facilitation of scene construction (Hassabis and Maguire, 2009). As our task included a series of one hundred unique scenes, each repeated twice, it may have induced hippocampal activity due to estimating the novelty of the scene, although this was not the subjects’ task. The individual hippocampal units seem to be highly selective in their responses, even within a category (Mormann et al., 2008), possibly explaining the lack of hippocampal activation revealed by many visual perception fMRI studies. The degree of discrimination of scenes from objects in the cluster S7 was the lowest one, significantly lower than of the cluster S3, between a little higher discrimination in the HIP region and lower in the ATC region.

Another scene-selective area in our experiment was the region along the anterior collateral sulcus, mostly comprising the anterior parahippocampal, fusiform, and entorhinal cortex. The channels in this area formed the S4 cluster and were dispersed over PHLG, FUG, HIP, and ATC regions. This area, together with the anterior hippocampus, was described to be more active in a scene recall task during correct judgments about scene novelty (Rombouts et al., 2001). Its activation in our experiment could, therefore, be connected to the novelty of half of the presented scenes and a weak familiarity with the other half.

The largest BGA responses were found in the S2 cluster containing five channels from three patients in the posterior lingual gyrus, at the junction of the collateral and lingual sulcus. Despite this large response, the discrimination of scenes from objects was rather low. The channels in this cluster were located more posteriorly than the most recent probabilistic localization of the PPA area (Weiner et al., 2018).

The last scene-selective area was localized around in the posterior precuneus (cluster S5). The precuneus activity in scene object discrimination could be associated with its role in spatial attention and its shifts (Cavanna and Trimble, 2006), which are more probably in spatial scenes than single objects without background. Besides, the precuneus is involved in spatial judgments using egocentric reference frames and translation between egocentric and allocentric coordinates (Byrne et al., 2007; Moraresku and Vlcek, 2020), which are also the processes more likely to occur when viewing spatial scenes than centered single objects.

### Areas Selective for Objects

Functional imaging studies defined the LOC as an area responding more strongly to photographs of everyday objects than shapeless textures (Malach et al., 1995). It covers a large area from the lateral OC to the posterior temporal regions, both ventral and lateral. Subsequently, it was subdivided into two areas discriminated by their functional properties (Grill-Spector et al., 1999). Using the fMRI adaptation paradigm, the authors showed that while the more posterior part (named LO) distinguishes between the same object being translated or transformed in size, the anterior portion in the fusiform gyrus (named pFs) preferentially displays position and size invariant responses.

The O1 and O3 clusters in our analysis were localized to a similar area. Cluster O1 was comprised of channels around the anterior occipital sulcus, in the middle and superior temporal, middle occipital, inferior temporal gyri, and also the temporo-occipital transition zone and posterior angular gyrus. It included mostly channels in the LTC region. It was, therefore positioned slightly more anteriorly than the fMRI localized LO area near the lateral occipital sulcus. Cluster O3 was localized more anteriorly, covering channels mostly in the posterior part of the fusiform gyrus, but also in the inferior temporal gyrus, corresponding to the pFs area. Channels in this cluster were dispersed mostly over the FUG, PHLG, and LTC regions. They showed superior object-scene discrimination, agreeing with its previously reported strong shape selectivity (Grill-Spector et al., 1999). An earlier human iEEG study demonstrated a BGA response selective for tools localized to a similar area (Vidal et al., 2010). A large number of object-selective channels in the O4 cluster were also positioned in a more anterior temporal area, comprising anterior parts of the parahippocampal gyrus, entorhinal, and perirhinal cortex, temporal pole, and also the inferior temporal gyrus (PHLG, LTC, and ATC regions). A similar area in the temporal pole responded to the familiarity of faces and scenes in an early PET study (Nakamura et al., 2000), suggesting its connection to recognition memory. Moreover, the perirhinal cortex seems to represent object-specific semantic information, as documented by an fMRI study (Clarke and Tyler, 2014). This study also showed the gradient of semantic specificity along the ventral stream, increasing anteriorly. Object recognition was also associated with brain activity in the anterior regions of the temporal lobe in another fMRI study (Bar et al., 2001). The contrast of successful to almost successful object recognition revealed activity in the anterior parahippocampal gyrus (besides activation of the LOC area), close to our O4 cluster. The same contrast also showed activity in the inferior frontal gyrus, which, according to an earlier publication, reflects the general effort, semantic analysis, and/or general feedback processes (Bar et al., 2001). We found several similarly localized object-selective channels in the O7 cluster. The activity of channels in these two clusters could reflect object recognition described in the above-mentioned studies. An important distinction between the objects and scenes in our test was that the objects were all familiar from everyday life, in contrast to the scenes, which were selected to be generally unfamiliar. Therefore, the results could reveal sites responding to familiarity instead of the object specifically.

Another important characteristic of all object stimuli in our test was that they could be grasped and manipulated by hand, as we excluded any pictures containing furniture or animals. This difference relative to the scene stimuli seems to manifest in the activity of brain areas related to tool use. Several such brain regions were revealed by an fMRI study, where subjects learned how to manipulate novel objects and were scanned during their visual presentation both before and after the training (Weisberg et al., 2006). This training increased activity in four areas: mainly the fusiform gyrus (LOC area), but also the middle temporal gyrus, the left intraparietal sulcus, and the left premotor cortex. These areas correspond to the location of object-selective channels in our data: the O3 cluster in the fusiform gyrus, mentioned above, and O5 in the posterior part of the angular gyrus. The areas around the intraparietal sulcus, mainly posterior, have been associated with object graspability. In one study, the activity in the posterior intraparietal sulcus was induced by the presence of both tools and graspable objects, relative to animals (Mruczek et al., 2013). An additional area, devoted to the execution and observation of tool action is the anterior supramarginal gyrus (Orban and Caruana, 2014), overlapping with the next cluster O6 in our data. In a meta-analysis of seven PET studies, tools activated the left posterior middle temporal region and to a lesser degree, the supramarginal gyrus (Devlin et al., 2002). In an earlier PET study (Grafton et al., 1997), passive viewing of familiar tools was connected with activation of the premotor cortex, and also the left inferior frontal gyrus, which formed the majority of channels in cluster O7 in our data.

A small cluster O2 of three channels also appeared in the orbitofrontal gyrus. The orbitofrontal cortex is known to be involved in reward learning and decision making (Rolls, 2004), but it was also shown to be activated by confidently identified visual objects bearing meaningful associations in humans (Chaumon et al., 2013). The orbitofrontal cortex also appeared in the contrast of recognized and unrecognized objects in an fMRI study (Bar et al., 2001).

### Temporal Scheme of Processing

Using the results of two complementary analyses of the temporal dynamics of scene and object discrimination, we can discuss the overall scheme of these two categories processing. We recorded the first discrimination of Scenes from Objects in the PPA (cluster S3 and the PHLG region) at 164 ms after the stimulus. It was also the longest one in our data, with the length of discrimination of 338 ms (or spanning for 400–500 ms according to the 100-ms time bin analysis). The onset was close to the latency of discrimination between buildings and non-building objects (170 ± 34 ms) seen in broadband gamma of a previous iEEG study focused on the PPA (Bastin et al., 2013b). The length of this effect was also similar to the length of discrimination in our data, lasting until about 550 ms and was also consistent with another intracranial EEG study documenting multiple processing stages in the PPA (Bastin et al., 2013a). The latency of response in our data was also similar to the onset of scene-selective responses previously observed in the parahippocampal LFP (Mormann et al., 2017). Higher stages of visual scene processing in the ventral cortex were estimated to occur at a similar time (141 ms) by a classification analysis on MEG data, although early visual areas discriminated individual scene images before 100 ms (Cichy et al., 2017). A more posteriorly located cluster S2 in the posterior lingual gyrus showed similarly early but shorter scene-object discrimination.

Next, the discrimination of scenes from objects appeared in several areas of both the ventral and dorsal visual streams. Ventrally, the cluster S4 near the anterior collateral sulcus showed only a short duration difference between scenes and objects, at 211 ms after the stimulus. Dorsally, scene-object discrimination appeared in two areas, first close to the transverse occipital sulcus near the OPA area (cluster S1) at 242 ms and second in the posterior part of precuneus (cluster S5) at 215 ms. The onset latency in cluster S1 was markedly longer than the onset of discriminated scene layout appearance in the OPA in an fMRI-MEG study (60 ms; Henriksson et al., 2019). According to this and another study (Kamps et al., 2016), the OPA is specialized in the discrimination of spatial boundaries (see also Julian et al., 2018). All our scene stimuli were mainly outdoor views of landscapes and buildings with indistinct spatial boundaries, possibly explaining the long latency of OPA scene-object discrimination. But, similarly, late responses, with a latency around 300 ms, were observed for scene presentation using MEG (Sato et al., 1999), with one of the sources estimated to be a parieto-occipital junction, close to our S1 cluster.

This time range over 200 ms is in agreement with the scalp EEG experiment focused on temporal dynamics of scene processing. The P2 component, peaking at 220 ms, was described as an ERP marker for scene processing (Harel et al., 2016), showing the earliest discrimination between scenes and both objects and faces. In a follow-up parallel ERP and fMRI study, this component was localized to the scene-selective areas, OPA and PPA (Kaiser et al., 2020). But surprisingly, cluster S3 in our data, localized to the PPA, showed earlier discrimination at 164 ms. Kaiser et al. (2020) also described earlier discrimination of spatially intact scenes starting at 55 ms and localized to V1, close to the time of discrimination of global scene properties at 84 ms in the Oz channel in scalp EEG study (Lowe et al., 2018). Results in this time range below 100 ms support conclusions from an earlier iEEG study showing decoding of five visual categories at around 100 ms after the stimulus (Liu et al., 2009). In the data set of the current study, none of the active channels was in the primary visual cortex.

At a later stage of scene processing, the cluster S6, encompassing the scene-selective MPA area, showed scene object discrimination at 307 ms, which lasted for the next 244 ms. The retrosplenial cortex showed higher activation for allocentric to egocentric processing at a close time interval from 350–650 ms in an intracranial EEG study (Bastin et al., 2013a). In the cluster S7 near the anterior hippocampus scene-object discrimination appeared with a similar onset of 326 ms. It was about 100 ms slower than in the more posterior clusters S2–4, which agrees with the differences in response latency reported for single-unit hippocampal activity (Mormann et al., 2008; Quiroga, 2012). The cluster with the fastest Objects from Scenes discrimination was O1 partially overlapping with the object-selective area LO, having an onset latency of 215 ms. This was noticeably longer than LFP latency reported in a similar region near the posterior portion of the inferior temporal sulcus in patients with subdural electrodes (73 ms; Yoshor et al., 2006). This study, however, reported the latency of a simple response onset, not an analysis of the difference between other types of stimuli. Besides, our cluster O1 included channels from two regions, with OC having a significantly shorter time of discrimination (153 ms) than the LTC region (263 ms). Therefore, the O1 cluster is not probably a functionally homogeneous unit.

The object scene discrimination appeared next in one ventral and two dorsal clusters. The ventral one was cluster O3 in the posterior fusiform gyrus, corresponding to the pFs area. The discrimination between object and scene activation in this cluster occurred later than in the O1 cluster (at 255 ms) and about 100 ms later than in the OC region. A similar timing was reported for a surface-negative potential with a peak latency of around 200 ms selective to objects found in the lingual, fusiform, and inferior occipital gyri in patients with subdural electrodes (Allison et al., 1999). The discrimination in the cluster O3 was also the longest one, according to the 100-ms time bins analysis, it lasted for 500–600 ms. The two dorsal clusters with a similar time of discrimination were O5 in the posterior part of the angular gyrus (257 ms) and O6 in the anterior supramarginal gyrus (291 ms). EEG and MEG studies recording from these areas have focused on the sources of the visual and auditory oddball task and localized here some of the generators of the P3 ERP component with a similar latency (Halgren et al., 1998; Brazdil et al., 2005).

At a later stage, with a significantly slower time of discrimination than the O1 cluster, three other clusters showed object scene selectivity. Cluster O4 (317 ms) was located around the anterior end of the collateral sulcus, while two clusters were in the FC, O7 (345 ms) near the inferior frontal sulcus and O2 (345 ms) in the orbitofrontal gyrus. These times are considerably longer than in a MEG study focused on top-down control of object recognition in the ventral cortex (Bar et al., 2006). In this study, the orbitofrontal LFP activity associated with object recognition peaked at 130 ms. This short latency may be explained by specific experimental design, with a very short masked object presentation for 26 ms and the repeated presentation of the same object.

In general, these processing schemes for scene and object recognition are in agreement with the concept of dorsal and ventral visual streams. Specifically, the high overlap in activations in individual areas, especially the long processing in the S3 (PPA) and O3 (pFs) clusters, overlapping in time with all other clusters, are consistent with the view of the visual pathway as a highly interactive and recurrent network (Kravitz et al., 2013). The late and prolonged discrimination of scenes in the S6 (MPA) cluster is in agreement with its position in the parieto-medial temporal pathway (Kravitz et al., 2011).

### Specificities of Our Experimental Design

Our study raises questions about the comparability of fMRI and electrophysiological experiments focused on visual image processing. The fMRI BOLD signal correlates with the local field potential signal optimally when in the BGA range (Mukamel et al., 2005), even though the correlation seems to depend on the cortex region (Conner et al., 2011). Both broadband gamma, in the range of 80–150Hz, and BOLD signal, seem to reflect inputs from neighboring neural circuits (Ojemann et al., 2009). However, our experiment, which used a simple oddball design, without any memory requirements, revealed more brain areas associated with scenes and object perception than any previously reported study using similar experimental designs. Scene responding channels appeared not only in the expected PPA, OPA, and MPA regions (Spiridon et al., 2006) but also in areas associated with scene novelty, such as the hippocampus and the anterior parahippocampal gyrus. Object responding channels were found in areas documented to be object-selective (both portions of LOC; Grill-Spector et al., 2000), as well as in areas associated with tool use (intraparietal sulcus, supramarginal gyrus, middle temporal cortex) and object recognition (inferior frontal and orbitofrontal gyri, perirhinal cortex). This disparity may be explained by the unique approaches of our study. First, we used a specific experimental setup with only outdoor scene stimuli, only graspable objects, and 100 unique stimuli from each category, each repeated twice. Future studies will be necessary to clarify this view. We also defined the object selectivity relative to scene stimuli, which is different from many functional imaging studies. Second, we evaluated the difference between the categories at all time points within the 800 ms long epochs of the 64 Hz BGA signal, and a significance noted at any time point (FDR corrected) resulted in the channel being identified as selective. This time precision is not possible in functional imaging and was not used in most iEEG studies (Bastin et al., 2013a,b; but see Mormann et al., 2017). Third, we used an event-related design with each block containing stimuli from all target categories, which is different from many functional imaging studies using blocks of single category stimuli.

Possibly, our results could be influenced by low-level spatial properties of visual stimuli. We used grayscale images with normalized average luminance and contrast, to prevent the potential effect of low-level properties of individual stimuli categories. However, both image categories still differed in some characteristics. In contrast to scenes, the object images contained a uniform background and presumably had a lower power of high spatial frequencies (SF). Responses of the scene- and object-selective areas are reportedly influenced by image SF, but to a different degree and with some controversy. According to some studies, the PPA seems to be more strongly activated by high SF (Rajimehr et al., 2011; Zeidman et al., 2012), but others report stronger responses to lower SF (Peyrin et al., 2004). This contradiction may be explained by another finding of the PPA being sensitive to the interaction between SF and image contrast (Kauffmann et al., 2015). In this study, PPA responded more to low SF, but it responded more to high SF under normalized contrast. According to Kauffmann et al. (2015), the effect of SF on the MPA region seems to be also dependent on the image contrast, while OPA was activated more by high SF independently on the image contrast. High SF was preferential for also the two object-selective areas (LO and pFs; Canário et al., 2016). Therefore, we argue that the effects observed in our study were connected to the semantic content rather than the low-level properties of the images.

### Study Limitations

Despite its merits, this study has several limitations. A disadvantage of intracranial recordings, in humans, is that coverage of the brain is inevitably limited. Although, we used data from 2,707 bipolar channels from 27 patients, the posterior OC was not implanted and while the coverage of the right temporal cortex was dense, it was much lower in areas such as the left frontal or parietal cortex. These regions may contain other category-selective channels that we could not investigate at this time.

The validity of the clustering method has several caveats. First, the clusters were computed from a limited number of category-selective channels with a highly varying density over the brain. Using data from a different group of implanted patients may result in a different set of clusters, with probably higher overlap with our clusters in densely implanted regions and low overlap in more sparsely implanted areas of the brain. With a higher channel density, some clusters could split while others could merge. Second, similarly to an earlier publication (Engell and McCarthy, 2014), we computed the clusters using channel MNI coordinates, which however do not take into account anatomical boundaries (sulci and fissures) or cortex gyrification. The use of cortical surface distances could change some channel-cluster assignments. Third, our approach using the absolute MNI “*x*” coordinates would not reveal any laterality in cluster localization. This approach enabled us to cluster the low number of category-selective channels and helped avoid false cluster laterality due to uneven channel distribution in both hemispheres. However, it could erroneously merge clusters with slightly distinct localization in the left and right hemispheres. Despite these limitations, we believe the clustering method we applied revealed valuable information about the distribution of scene and object selectivity in the brain.

The data were collected from epilepsy patients and there is a possibility they could reflect inherent pathological conditions. However, trials showing any type of epileptiform activity were discarded for every channel and “epileptic” channels did not display a different response magnitude or time from non-epileptic channels. Thus, we believe that our data reflect primarily physiological mechanisms.

## Conclusions

Here, we describe and characterize the electrophysiological activity in areas selective for scenes or objects over many cortical regions by direct iEEG recording. We confirm the scene and object response selective PPA and LOC areas, consistent with reports from functional imaging, and extend the previous iEEG reports of two scene-selective areas: the MPA and OPA. Moreover, our results extend this network into other brain areas, which have not yet been described by iEEG. Selective processing for scenes was apparent in parts of the anterior temporal lobe, associated with scene novelty, such as the hippocampus and the anterior parahippocampal gyrus. Also, selectivity for objects appeared in areas associated with tool use, the intraparietal sulcus, supramarginal gyrus, and middle temporal cortex, as well as areas connected with object recognition, such as the inferior frontal gyrus and the perirhinal cortex. By the detailed analyses of the time course of category selectivity, we document its progress through the dorsal and ventral visual streams. The high overlap in the time of processing is consistent with the view of the visual pathway as a highly interactive network. Consequently, the main contribution of this study is our description of direct electrophysiological activity selective for scenes and objects in numerous areas of the human brain. Future studies could address the functional connectivity between these areas to shed further light on the novel network dynamics of the brain during visual perception.

## Data Availability Statement

The raw data supporting the conclusions of this article will be made available by the authors, without undue reservation.

## Ethics Statement

The studies involving human participants were reviewed and approved by Ethics Committee of Motol University Hospital (02-APR-2014). Written informed consent to participate in this study was provided by the participants’ legal guardian/next of kin.

## Author Contributions

KV, PM, and JH conceptualized the study. KV and IF developed the test. KV, RJ, PJ, LH, and JH implemented data analysis techniques. KV, IF, and TN conducted the experiments with the help of colleagues listed in the “Acknowledgments” section. MT, AK, PK, and PM verified the neuroanatomy localization. KV analyzed the data and created figures with the help of IF, LH, and PJ. KV wrote the manuscript with the help of PM, JH, IF, LH and TN. All authors contributed to the article and approved the submitted version.

## Conflict of Interest

The authors declare that the research was conducted in the absence of any commercial or financial relationships that could be construed as a potential conflict of interest.

## Abbreviations

ATC: anterior temporal cortex brain region
AUC: the area under the curve
BGA: broadband gamma activity
CC: cingulate and paracingulate cortex brain region
FC: frontal cortex brain region
FDR: false discovery rate
FLPG: fusiform, lingual and parahippocampal gyri brain region
HIP: hippocampus brain region
iEEG: intracranial electroencephalography
INS: insula brain region
LOC: lateral occipital complex
LO: a lateral occipital portion of LOC
LTC: lateral temporal cortex brain region
MPA: medial place area
OC: occipital cortex brain region
OPA: occipital place area
PC: parietal cortex brain region
pFs: posterior fusiform sulcus portion of LOC
RSC: retrosplenial cortex and precuneus brain region
PPA: parahippocampal place area
SF: spatial frequency.
